# Supplementary material for: Structure and thermodynamics of supported lipid membranes on hydrophobic van der Waals surfaces
Source: Soft Matter. 2024 Jun 27;20(29):5724–32. doi: 10.1039/d4sm00365a (PMC11268427; doi:10.1039/d4sm00365a)
Supplement: SM-020-D4SM00365A-s001 [file SM-020-D4SM00365A-s001.pdf]

Electronic Supplementary Material (ESI) for Soft Matter.
This journal is © The Royal Society of Chemistry 2024

Electronic Supplementary Information

Structure and thermodynamics of supported lipid membranes on hydrophobic van der Waals surfaces

Harriet Read, Simone Benaglia, Laura Fumagalli

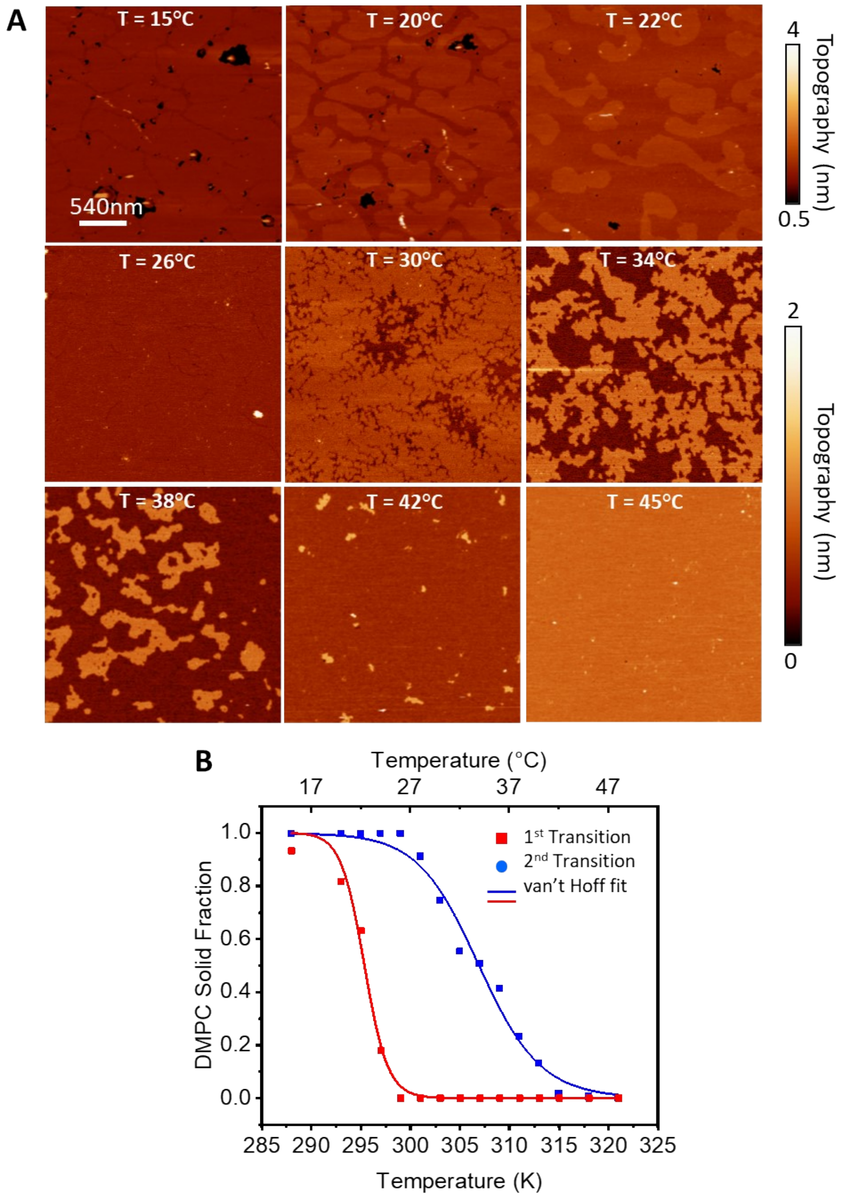

S1 Phase transition of DMPC SLBs on mica and silicon

Figure S1. DMPC vesicles were formed in pure water following the same protocol in the main text and previous literature.[1] A complete DMPC supported lipid bilayer with small defects formed on freshly cleaved mica, using 0.2 mg/ml DMPC/DI suspension. (a) The temperature-dependent phase transition of the bilayer was tracked using AFM. Increasing the temperature led to a decoupled leaflet transition, with the top leaflet (lipid layer facing the aqueous solution) transitioning from the gel ordered to fluid disordered phase. Once this transition was completed, the second, lower leaflet (adjacent to the substrate) made the transition. The temperature was increased until all parts of the bilayer were transitioned. (b) To analyse the transition temperature of each leaflet separately, the fraction of the gel phase vs temperature was plotted and fitted using van't Hoff analysis. This revealed a transition temperature of the upper/lower leaflet as 22 °C (295 K) and 34 °C (307 K), respectively. The second

transition is much higher than the expected transition temperature (determined using scanning differential calorimetry) due to the interaction between the substrate and the lipids.[2]

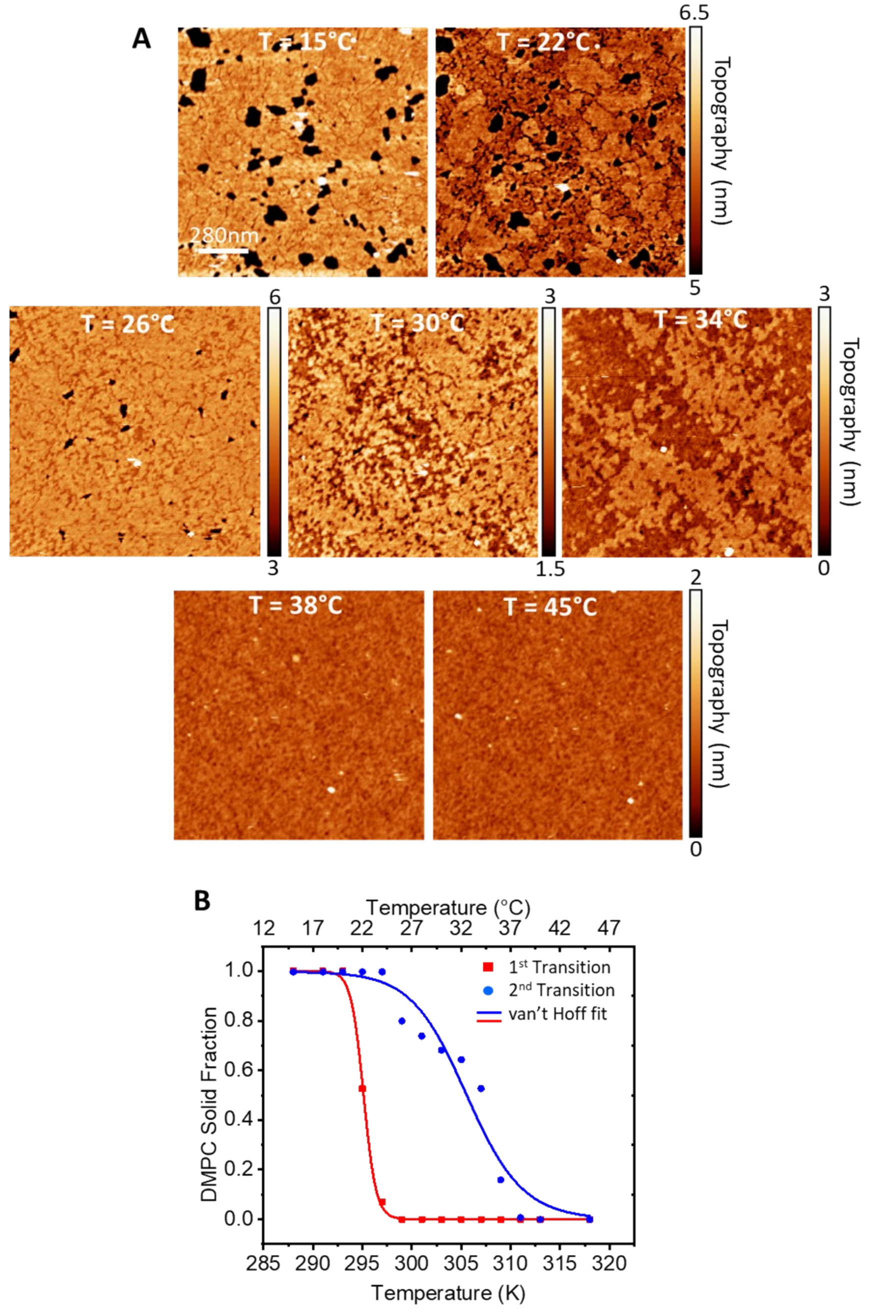

Figure S2. A complete DMPC supported lipid bilayer with small defects formed on clean silicon wafers using 0.2 mg/ml DMPC/DI suspension. (a) The temperature-dependent phase transition of the bilayer was tracked using AFM in liquid environment. Increasing the temperature led to a decoupled leaflet transition, with the top leaflet (lipid layer facing the aqueous solution) transitioning from the gel ordered to fluid disordered phase, as was seen on mica. (b) To analyse the transition temperature of each leaflet separately, the fraction of the gel phase vs temperature was plotted and fitted using van't Hoff analysis. This revealed a transition temperature of the upper/lower leaflet as 22 °C (295 K) and 32 °C (307 K), respectively. The transition temperature of the first leaflet is very close to the one extracted on mica and

those found via DSC. The second transition is higher than the expected transition temperature due to the interaction between the substrate and the lipids.

S2 Phase transition of DMPC monolayer on HOPG

To compare the results obtained on HOPG with those on mica and silicon, the fraction of the S_o/L_d phase was estimated from the monolayer height change on HOPG (Figure 3a). The distributions in Figure S3b, as the cross sections in Figure 3 of the main text, show that the average height of the monolayer tends to decrease upon increasing the temperature. The change in height with respect to the temperature is plotted in Figure 3c. To convert the change in height into fraction of the S_o, we assumed that the average height of the membrane at a temperature T, H(T), is given by the sum of height in the S_o, h_S, and L_d, h_L, phase normalised by the fraction of the membrane in the S_o, f_S, and L_d, f_L, phase respectively such that: $H(T) = h_S f_S + h_L f_L$. Considering that the sum of the fraction of the membrane in the S_o and L_d phase may be written as $f_S + f_L = 1$, by simply rearranging for f_S we obtain the following expression : $f_S = (H(T) - h_L)/(h_S - h_L)$. This allows a plot of the S_o fraction vs temperature as we did for the mica

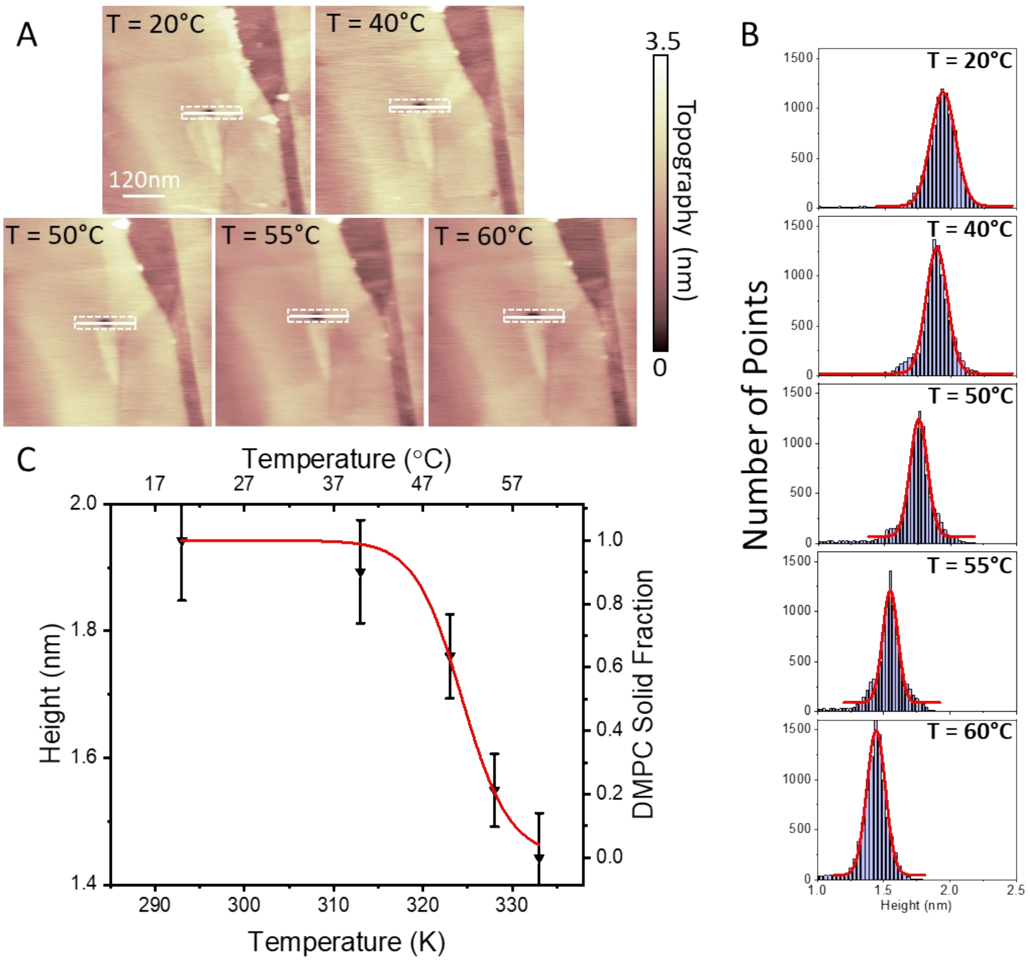

and silicon substrates. The curve can be seen in Figure S3c and in Figure 3 of the main text.

Figure S3. DMPC supported lipid monolayer formed on HOPG (0.1mg/ml DMPC/DI suspension). (a) A defect in the monolayer was used to measure the thickness of the monolayer with increasing

temperature. (b) Height distributions show the decrease in monolayer thickness from 1.8 nm at 50 °C to 1.4 nm at 60 °C, indicating a phase transition from S_o to L_d. (c) Temperature dependence of the membrane height (left axis) and fractional occupancy of the DMPC S_o phase (right axis) - where the error bars represent the uncertainty in the height measurement.

S3 Morphological features of DMPC and DLPC on HOPG and hBN

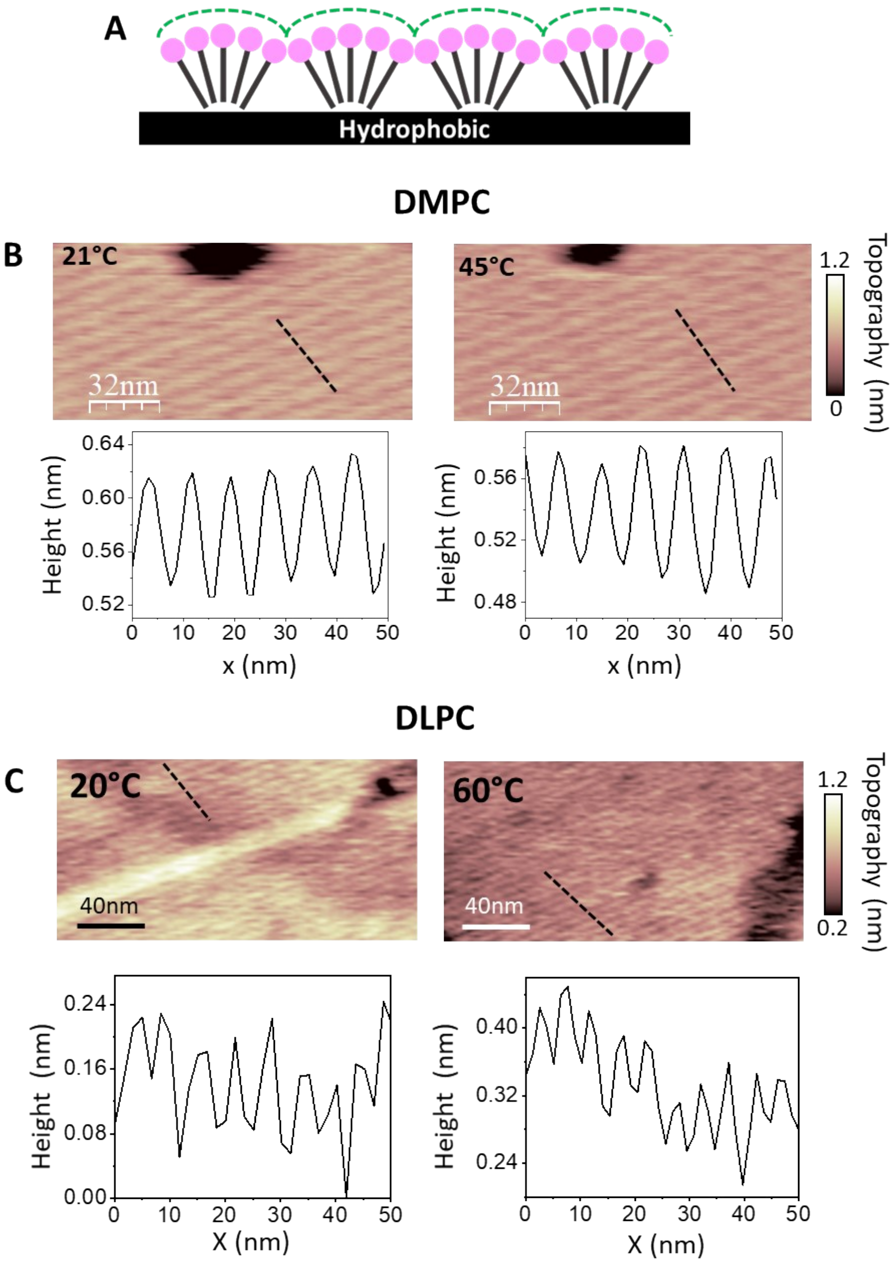

Figure S4. Morphology of zwitterionic lipid membranes formed on HOPG. The lipid monolayer shows stripe-like features, indicative of a hemimicellar conformation (a). Similar features were recorded for both DMPC (b) and DLPC (c). Importantly they were stable and did not change significantly with temperature.

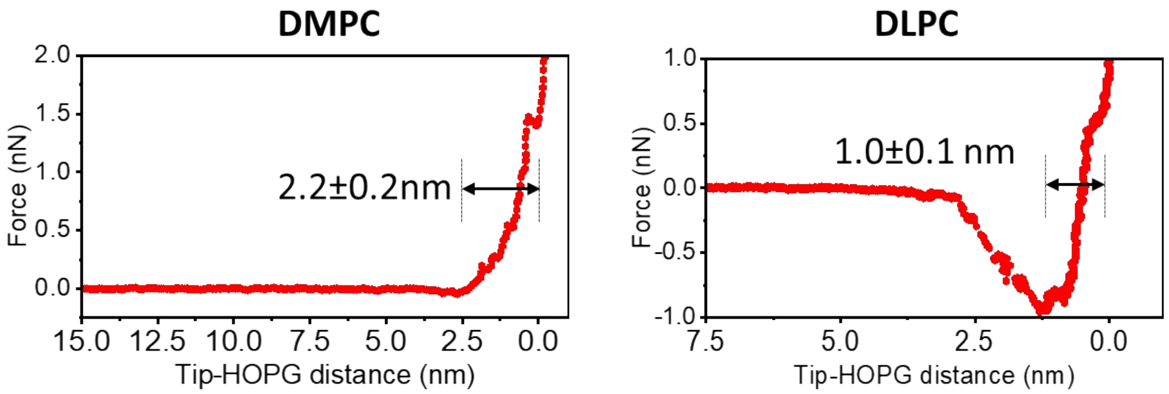

Figure S5. Force distance curves (FDC) obtained on lipid membranes formed on HOPG for both DMPC and DLPC. The breakthrough event into the lipid monolayer allows to determine the thickness of the lipid membrane. The FDCs were obtained with a loading rate of 150 nm/s and 20 nm/s for the experiments on DMPC and DLPC, respectively.

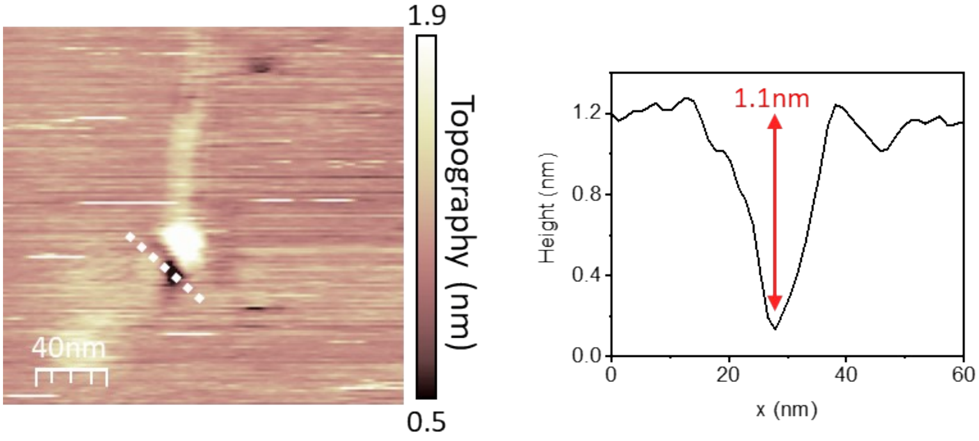

Figure S6. DLPC supported lipid membrane on HOPG. Topography map showing a defect in the lipid membrane from which a cross section was taken to determine the thickness of the membrane, which is consistent with the presence of a single monolayer adsorbed at the surface of HOPG.

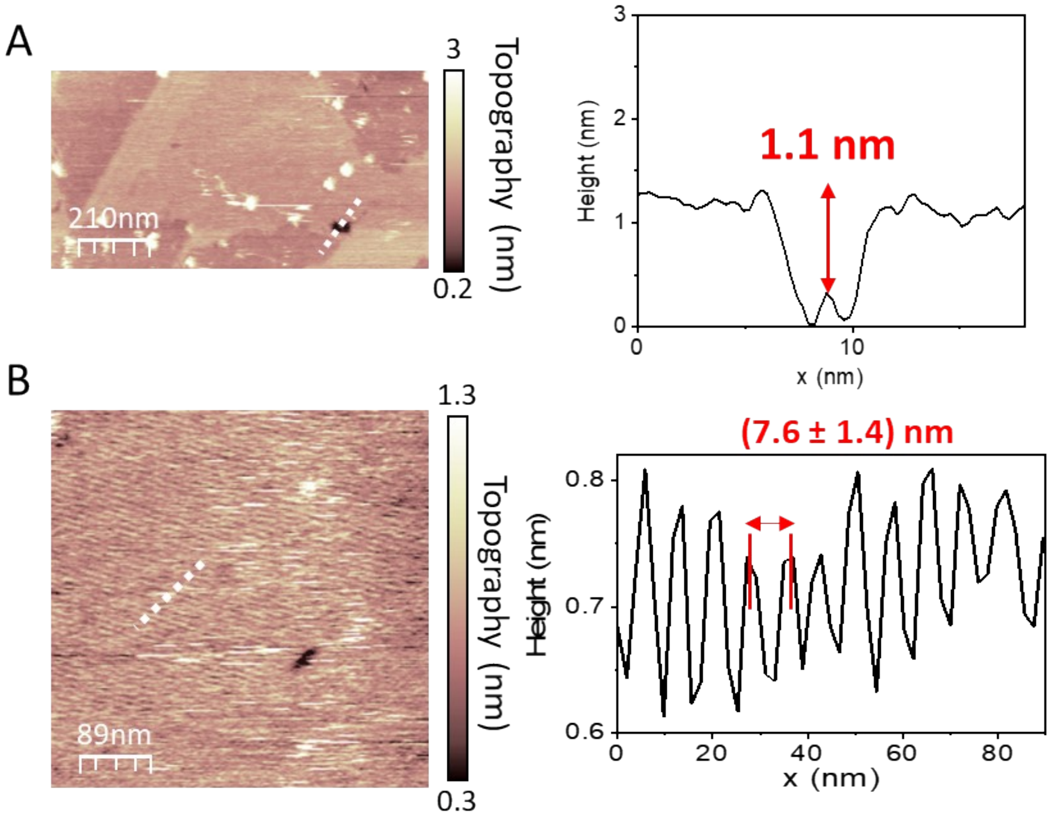

Figure S7. (a) Topography map showing a fully formed DLPC supported lipid membrane on h-BN at T = 7 °C (gel phase). A defect in the membrane was used to determine a membrane thickness of ~1.3 nm. The thickness agrees with that found on HOPG. (b) Topography map showing that like on HOPG, stripe-like structures in the membrane form. The periodicity of the ripples was measured to be around 7.5 nm.

S4 Phase transition of DLPC monolayer on HOPG

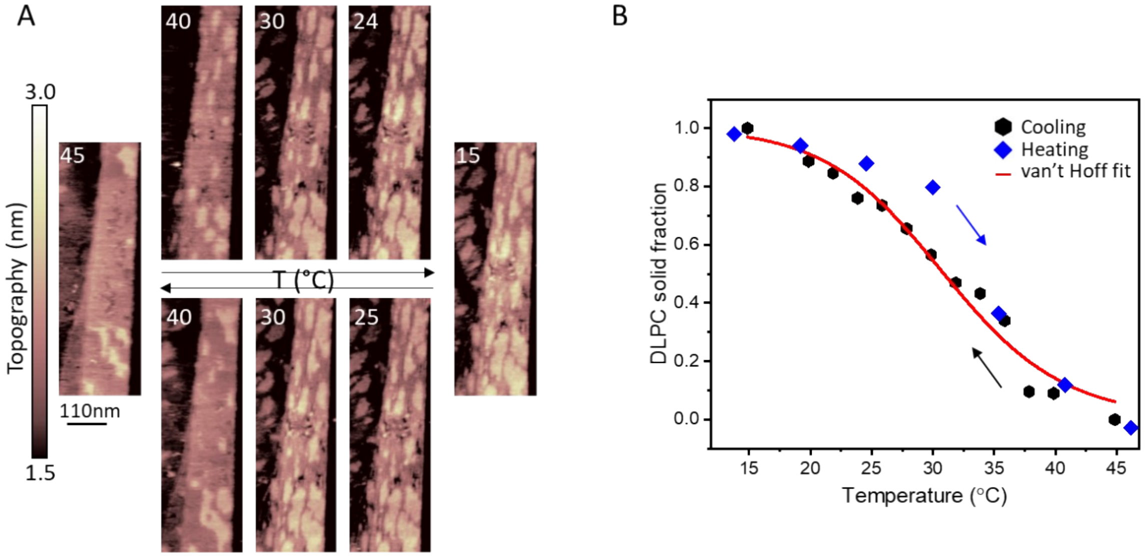

Figure S8. (a) AFM topography images as function of temperature (upon cooling and heating) of a DLPC membrane on HOPG. Taller domains appear with decreasing temperature (reversible process), indicating an equilibrium phase-transition. (b) The van't Hoff analysis was on the experimental data obtained during cooling, revealing a transition temperature of DLPC monolayer on HOPG of $T_m \approx 30.9$ ◦C.

References

[1] S. Attwood, Y. Choi and Z. Leonenko, *Int. J. Mol. Sci.*, 2013, **14**, 3514–3539.

[2] H. M. Seeger, A. Di Cerbo, A. Alessandrini and P. Facci, *J. Phys. Chem. B*, 2010, **114**, 8926–8933.

[3] H. Bi, X. Wang, X. Han and K. Voïtchovsky, *Langmuir*, 2018, **34**, 9561–9571
